# Legacy contamination in Chicago urban park soils: Spatial patterns, socioeconomic disparities, and implications for urban agriculture

**DOI:** 10.1016/j.isci.2026.116298

**Published:** 2026-06-11

**Authors:** Eriell M. Jenkins, Holly Heafner, James Montgomery, Victoire Soumano, Anna A. Paltseva

**Affiliations:** 1 Delta Urban Soils Laboratory, School of Geosciences, University of Louisiana at Lafayette, Lafayette, LA 70504, USA; 2 Department of Environmental Science and Studies, DePaul University, Chicago, IL 60614, USA; 3 Departments of Agronomy and Horticulture and Landscape Architecture, Purdue University, West Lafayette, IN 47907, USA

**Keywords:** soil science, environmental science, pollution, urban planning

## Abstract

Urban parks are increasingly used for recreation and urban agriculture, yet their soils may retain legacy contamination. This study quantified seven trace elements in topsoils of five Chicago parks (*n* = 233) using portable X-ray fluorescence. Pb, As, Cu, Zn, and Cr exceeded Illinois background levels in 89%–100% of samples; As and Pb exceeded EPA screening levels in 94% and 7%. Higher contamination was observed at parks farther from the city center: Douglass and Humboldt parks, in predominantly minority, lower income neighborhoods, versus Cottontail, Grant, and Webster. Principal-component analysis (PCA) confirmed an anthropogenic signature (PC1, 51.7%) driven by Pb, Zn, Cu, and As and a geogenic signature (PC2, 17.0%) associated with Mn and Ni. Areas in Douglass and Humboldt parks were classified as not safe for gardening in native soil; Webster as safe with minor restrictions. Park soil contamination follows socioeconomic gradients, reflecting environmental injustice and the need for targeted remediation.

## Highlights

• Pb, As, Cu, Zn, and Cr exceed Illinois background levels in 89%–100% of park soils
• Lower income, higher minority parks have higher Pb, Cu, Zn, As, and Cr
• Douglass Park classified as very highly contaminated by modified contamination index

## Graphical abstract

**Figure:**
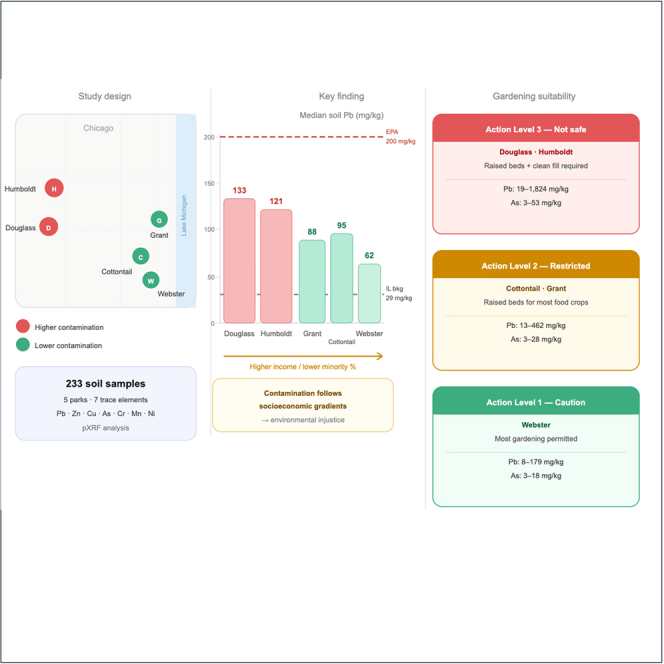

## Introduction

### Trace element contamination in urban parks

Urban parks and playgrounds are vital community resources that increasingly serve as platforms for community-led food production, contributing to public health, ecosystem services, and economic resilience^1^^,^^2^^,^^3^ Yet due to rapid urbanization and historical industrial legacies, these same spaces often function as significant reservoirs for trace element contamination, posing persistent risks to human and environmental health.^4^^,^^5^ Sources of this contamination are multifaceted, ranging from legacy inputs such as leaded paint and gasoline.^6^^,^^7^ improper industrial waste disposal, and emissions from metal processing and chemical industries,^8^^,^^9^^,^^10^^,^^11^ to modern anthropogenic contributions including traffic-related emissions, brake and tire wear, degradation of road materials, agricultural chemical applications,^12^ and leaching of preservatives from treated wooden structures.^13^

### Human health risks and exposure pathways

Human exposure to these contaminants occurs primarily through three pathways: direct ingestion of soil and dust, inhalation of resuspended particulate matter, and dermal absorption.^7^^,^^13^^,^^14^^,^^15^^,^^16^ Contaminants may also enter the body through food or water from affected soils.^17^ Children represent the most vulnerable population due to their higher respiratory rates, developing physiological systems, and characteristic ground-level play and hand-to-mouth behaviors, making soil and dust ingestion the dominant exposure pathway.^18^^,^^19^^,^^20^ Excessive trace element accumulation is toxic, carcinogenic, and harmful to vital organs, with childhood exposures linked to developmental and learning disorders.^21^ Mielke et al.^22^ demonstrated this connection in New Orleans, finding a strong correlation between soil Pb levels and children’s blood Pb concentrations, with high-risk areas showing 6-fold greater contamination alongside elevated Zn and Cd.

Beyond human health, trace elements degrade soil quality by reducing fertility and disrupting microbial communities, and their non-degradable nature allows them to persist, contaminating water bodies via runoff and leaching and entering the food chain through crops grown in affected soils.^23^^,^^24^ Importantly, however, many trace elements are insoluble and not bioavailable, meaning total concentrations may overestimate actual toxicity.^25^^,^^26^ While non-carcinogenic risks in parks are often reported within acceptable thresholds, the persistence of neurotoxic and carcinogenic elements such as Pb and Cr(VI) necessitates proactive soil monitoring and site-specific assessment^6^^,^^13^^,^^14^^,^^15^^,^^16^—particularly as cities like Chicago explore expanding urban gardening activities into park spaces, where understanding trace element variability is essential to ensuring safe food production.

### Urban agriculture and soil quality in Chicago

The Chicago metropolitan region has a long and rich history of urban agriculture, dating from the early 1900s. During both world wars, the US government and civic groups advocated the creation of vegetable gardens in backyards and public spaces, including parks. During World War II, Chicago led the nation in wartime food production, with over 1,500 community gardens and 250,000 home gardens, many of which were established in city parks as part of the “victory garden” movement.^27^

In recent years, Chicago has experienced sustained expansion of urban agriculture, growing from scattered community gardens to over 890 documented growing sites by 2021, catalyzed by the city’s 2011 Urban Agriculture Zoning Ordinance that permitted community gardens and urban farms across most zoning districts as a matter of right (https://www.auachicago.org/). The Chicago Park District alone now hosts over 90 community gardens, including edible allotment plots, children’s learning gardens, and the Harvest Garden program, while NeighborSpace preserves approximately 120 sites as permanent urban greenspace (https://www.chicagoparkdistrict.com/facilities/community-gardens). As urban agricultural initiatives continue to grow, including the use of city parks for food production, it is important to evaluate the ability of soil to support healthy and sustainable agriculture. Despite the vast literature on urban soil contamination, localized studies are essential to understand site-specific risks.

Chicago, with its extensive industrial legacy, provides an important case where trace element contamination has been documented across multiple land uses.^28^^,^^29^ At the citywide scale, an investigation of 1,750 soil samples found a median Pb concentration of 217 mg/kg, over ten times the natural background, with 54% of samples exceeding the EPA’s 200 mg/kg residential screening level and hotspots reaching 6,284 mg/kg, concentrated on the west and south sides of the city.^6^ Site-specific studies reveal considerable variability depending on land use and management. In a Chicago nature preserve, Pb, Cu, and Zn levels reached 160, 130, and 320 mg/kg, respectively, likely from atmospheric deposition and runoff.^28^ In urban community gardens, Pb ranged from 10 to 889 mg/kg, with raised beds showing lower levels due to cleaner soil inputs, while adjacent bare areas exhibited higher concentrations.^30^ By contrast, a study of 21 urban gardens and farms found Pb, As, and Zn generally below concern levels, suggesting that management practices can help maintain soil health despite historical contamination.^31^

### Environmental justice and socioeconomic gradients

These contamination patterns intersect with deep inequities in park access and reflect deeply entrenched systemic racism characterized by economic and power imbalance, racial and class inequality, restrictive zoning laws (e.g., redlining), and past land use decisions that concentrated toxic industries and environmental burdens in racially, ethnically, and economically segregated areas of the city.^32^^,^^33^^,^^34^^,^^35^ This pattern is not unique to Chicago. Across eight cities in six Southern states, soil metal concentrations were significantly associated with minority and poverty rankings, with every ten percentile increase in minority rank corresponding to a 13.5% increase in soil As and a 10.6% increase in soil Pb.^36^ In Santa Ana, California, census tracts with median household incomes below $50,000 had five times higher soil Pb concentrations than high-income tracts,^37^ and in New Orleans, median household income declined from $45,500 to $24,000 as soil Pb concentrations rose.^38^ A review of 49 empirical studies further documented that low-income and minority populations consistently have access to fewer acres of park per person and to parks of lower quality and maintenance than more privileged populations.^39^

In Chicago specifically, there is disproportionate industrial pollution on the south and southeast sides, characterized by large concentrations of heavy industry and toxic waste facilities in predominantly Black and Latino neighborhoods. These areas are known as “sacrifice zones,”^35^ and over 60% of industrial sites are located in Chicago’s most socially vulnerable areas.^40^ Parks are high-contact environments where children may spend substantial time outdoors, often on exposed bare soil.^6^ Parks and their inhabitants within sacrifice zones often face higher environmental risks, making soil quality a public health and environmental justice concern. Thorstenson et al.^6^ demonstrated this connection directly, showing that soil Pb is an independent predictor of elevated childhood blood lead levels beyond socioeconomic factors, with an estimated 27% of children citywide at risk of exceeding the Centers for Disease Control and Prevention (CDC) reference value of 3.5 μg/dL based on soil exposure alone—rising to 57% in the most affected community areas on the west and south sides.

### This study

The primary goal of this study is to quantify trace element concentrations and map their spatial distribution in five Chicago’s urban parks, with the aim of evaluating their suitability for potential urban agriculture and safe public use. Urban parks are critical green spaces where direct human exposure to soils occurs through recreation, landscaping, and, increasingly, informal or planned food production. However, these environments may retain legacy contamination from historical industrial activities, traffic emissions, and the use of anthropogenic fill materials. We hypothesize that Chicago parks located closer to the city center and in historically industrialized areas will exhibit higher trace element concentrations due to cumulative anthropogenic inputs. To address this, we established the following objectives: (1) quantify concentrations of Pb, Cu, As, Mn, Ni, Zn, and Cr, along with key soil properties (pH, salinity, and organic matter), in soils from five representative urban parks; (2) evaluate how trace element concentrations vary in relation to proximity to the city center, historical land use, and surrounding neighborhood characteristics; (3) assess whether selected areas within parks meet safety thresholds for potential urban agriculture and human exposure; and (4) identify management and remediation strategies for locations with elevated concentrations.

## Results

**Figure 1:**
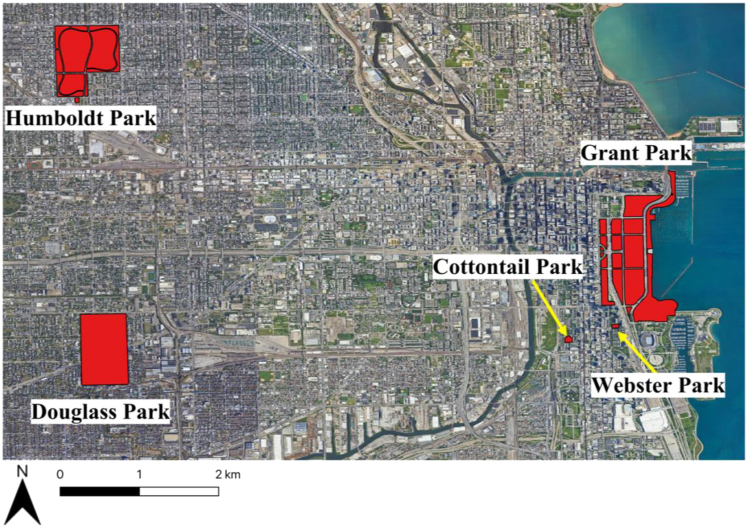
Soil sampling study sites in Chicago, Illinois (*n* = 233 samples)

Five Chicago urban parks were selected for this study, ranging in size from 1.2 to 313 acres, founded between 1844 and 2000, and representing residential and commercial community contexts (Figure 1; Table 1). Previous land uses varied from wetland and flat prairie to rail yards and unincorporated land, with soils classified predominantly as anthropogenic Udorthents and urban land-Orthents, reflecting extensive human modification of the natural soil landscape.

**Table 1:** Characteristics of the Chicago parks included in the study

Park name	# of samples	Size (acres)	Previous land use	Founded	Community type	Soil series
Webster	9	1.2	unincorporated	2000	residential	urban land-Orthents
Cottontail	12	2.35	rail yards	1990	residential	urban land-Orthents
Douglass	91	162	wetland	1869	residential	Elliot silt loam (fine, illitic, mesic Aquic Argiudolls); Anthroportic Udorthents
Humboldt	78	207	flat prairie	1869	commercial/residential	Bryce silty clay (fine, mixed, superactive, mesic Vertic Endoaquolls); Anthroportic Udorthents; urban land-Orthents clayey complex
Grant	43	313	unincorporated	1844	commercial	Orthents (entisol)

The soil series information was sourced from the Web Soil Survey.

### Soil pH, EC, and organic matter content

To contextualize trace element variability across parks, we first examined key soil properties that influence metal mobility and retention. Soil pH ranged from 6.86 to 8.26 across all samples (overall median 7.46) (Table S1). Douglass Park exhibited the greatest within-park variability (6.86–8.22), while Webster Park showed the most uniform pH (7.40–7.59). Electrical conductivity (EC) ranged from 72 to 702 μS/cm across all samples (overall median 220 μS/cm). The maximum value of 702 μS/cm was recorded at Douglass Park as an isolated observation; the park median was 274 μS/cm. Organic matter content ranged from 6.3% to 17.6% across all samples (overall median 9.6%). Douglass Park showed the widest within-park range (6.5%–17.6%), with the overall maximum of 17.6% recorded within that park.

### Trace element concentration patterns across parks

**Figure 2:**
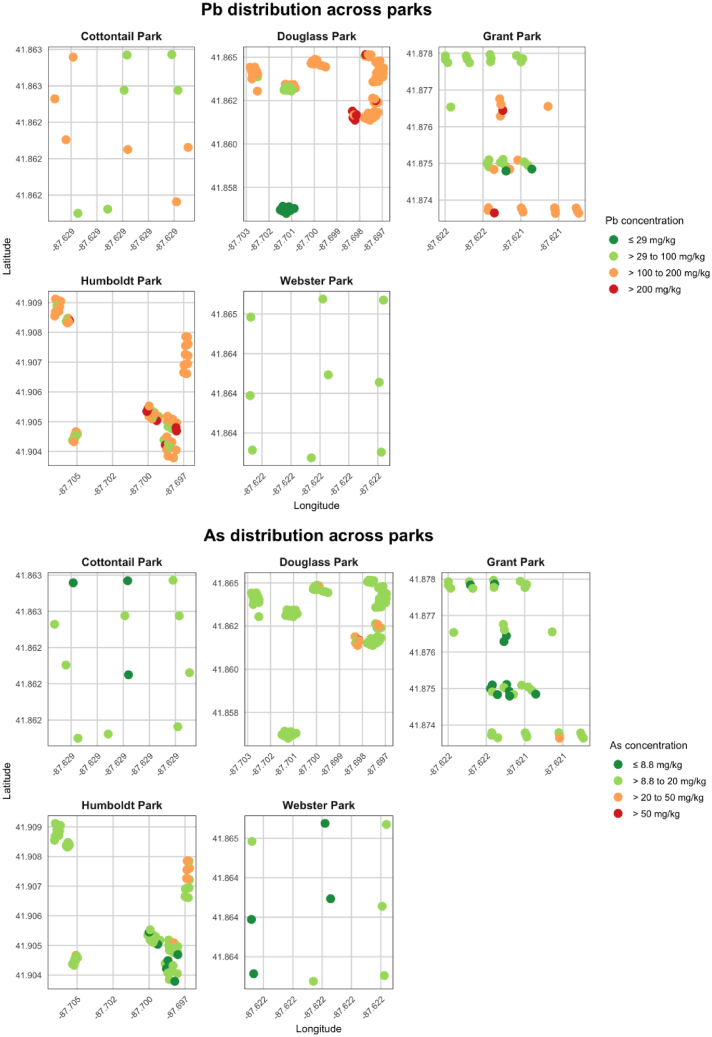
Spatial distribution of trace metal concentrations across five urban parks in Chicago Points represent individual soil sampling locations.

**Figure 3:**
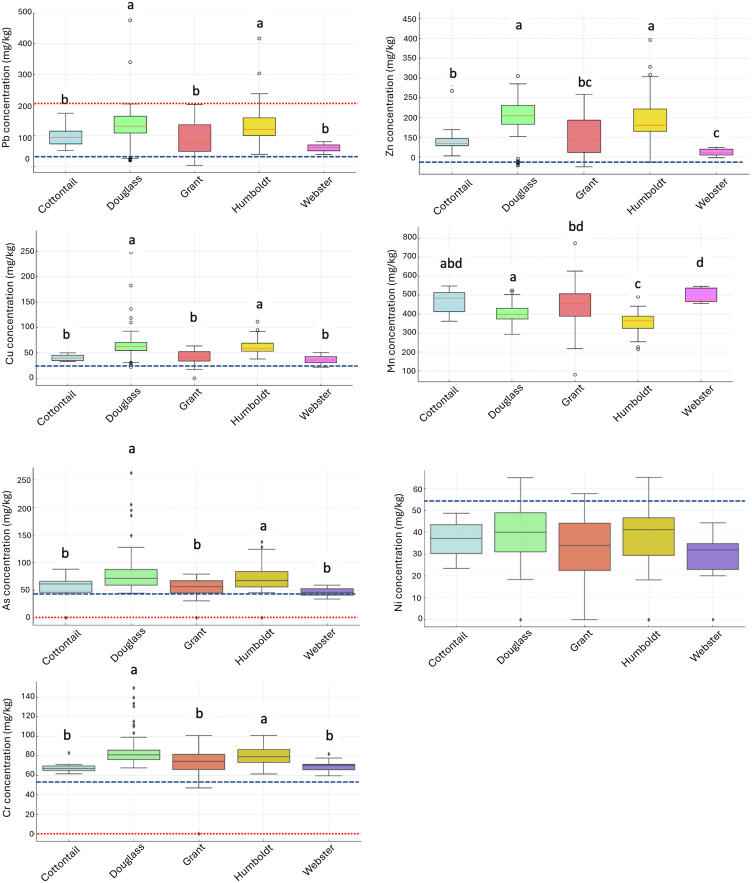
The concentrations of trace elements in Chicago urban parks The lines within the boxes represent the median for each park’s sample set, while the dots represent outliers in the data. Outliers above 500 mg/kg for Pb were omitted to improve figure readability. The red line spanning various graphs represents that element’s screening level (Table 3), while the blue line represents the background level of Illinois. When a red line was absent in the plot, the screening level was higher than the range of the graph’s concentration values. A regional background level was not found for Mn. Lowercase letters indicate a statistically significant difference between parks.

Trace element concentrations varied substantially across the five study parks, with consistent spatial gradients observed for most metals (Figures 2 and 3; Figures S1–S5; Table S1). Median concentrations of Pb, Cu, Zn, and As followed a clear ranking of Douglass Park > Humboldt Park > Grant Park ≥ Cottontail Park > Webster Park, with Douglass Park recording median values of 133, 63, 207, and 14 mg/kg for Pb, Cu, Zn, and As, respectively—2.1×, 1.8×, 1.9×, and 1.6× higher than the lowest ranked Webster Park (62, 36, 111, and 9 mg/kg). Cr followed a similar gradient (Douglass 81 > Humboldt 79 > Grant 74 > Webster 70 > Cottontail 67 mg/kg), though the range across parks was comparatively narrow.

Manganese departed markedly from this pattern. Median Mn was highest at Cottontail Park (485 mg/kg) and Webster Park (468 mg/kg) and lowest at Humboldt Park (364 mg/kg). This inverse relationship between Mn and the contamination gradient observed for the other metals suggests a geogenic rather than anthropogenic source for Mn. Nickel showed a distinct distribution: Humboldt Park recorded the highest median Ni (41 mg/kg), followed by Douglass Park (40 mg/kg), while Webster Park had the lowest (32 mg/kg).

For Pb and Zn, mean concentrations at Douglass Park (181 and 260 mg/kg, respectively) exceeded the corresponding medians (133 and 207 mg/kg) by 36% and 26%, indicating positive skewness driven by a localized contamination hotspot identified in the northwestern sector of the park. At all other parks, mean and median values were within 15% of one another, indicating approximately symmetric distributions without influential outliers (Table S1).

Figure 3 illustrates significant variations in trace metal concentrations across parks using Dunn’s test (*p* < 0.05) with Bonferroni correction. Douglass and Humboldt show the highest median Pb, Cr, As, Zn, and Cu concentrations, while statistical comparisons indicate significant differences between these two parks and other locations. Ni shows no statistically significant difference. Cottontail, Grant, and Webster show significant differences from Douglass and Humboldt. Overall, parks can be divided into two groups: (1) Douglass and Humboldt parks and (2) Cottontail, Grant, and Webster, exhibiting statistical similarities for many metals.

### Trace element levels relative to distance from the city center and guideline values

Among the five parks studied, samples located farther from the city center (Figure 1), defined as the intersection of State Street and Madison Street (41.882°N, 87.628°W) (a conventional reference point historically recognized as the origin of Chicago’s street grid numbering system), tended to have higher median concentrations of Pb, Cu, Zn, As, and Cr. Douglass Park (6.03–6.73 km from the city center) and Humboldt Park (6.29–7.16 km) recorded the highest median values for these elements, while Webster Park (2.00–2.03 km), Cottontail Park (2.15–2.20 km), and Grant Park (0.68–1.09 km) recorded lower values (Figures 3 and S6). Post hoc comparison confirmed that Douglass Park concentrations of Cu and Zn were significantly higher than all other sampling locations and that Douglass and Humboldt Parks formed a statistically distinct group from the remaining three locations for Pb, As, and Cr (Figure 3). Manganese did not follow this pattern, with no consistent relationship between Mn concentration and distance from the city center (Figures 3 and S6). Nickel showed no statistically significant differences among the parks, with all park medians falling below the Illinois geochemical background level of 53 mg/kg. Because samples were collected from five discrete locations rather than continuously distributed across a distance gradient, the observed tendency cannot be interpreted as a continuous spatial trend.

Table 2 highlights the extent of trace element contamination in Chicago park soils compared to EPA soil screening levels and Illinois geochemical background levels. Lead exceeded Illinois background levels in 95% of samples and EPA levels in 7%. Arsenic exceeded background and EPA levels in 89% and 94% of samples, respectively. Copper, Mn, Ni, and Zn rarely exceeded EPA thresholds but often surpassed background levels. Total Cr concentrations determined by pXRF exceeded the EPA screening level of 0.3 mg kg^−1^ at all sampling locations. It is important to note that this screening level is defined for hexavalent chromium [Cr(VI)], while pXRF measurements represent total Cr and do not differentiate between oxidation states. Because Cr(III) is the dominant and less toxic form in most soils, these results should be interpreted cautiously, and exceedance of the screening level cannot be directly attributed to Cr(VI) without speciation analysis.

**Table 2:** Percentage of Chicago park soils (*n* = 233) exceeding EPA soil screening levels and regional geochemical background levels for trace elements in Illinois soils based on Illinois Geological Survey in mg/kg

Element	Mean	Range	EPA (% above)	Bkgrd (% above)
Pb	146	4–1,820	200 (7)	29 (95)
As	14	<7–53	0.68 (94)	8.8 (89)
Cu	59	<15–279	3100 (0)	28 (97)
Mn	405	81–823	1,800 (0)	–
Ni	36	<30–65	820 (0)	53^a^ (9)
Zn	208	76–2,259	23,000 (0)	74 (100)
Cr	79	<30–149	0.3 (100)	56 (98)

a Max concentration.

### Principal-component analysis of trace elements

**Figure 4:**
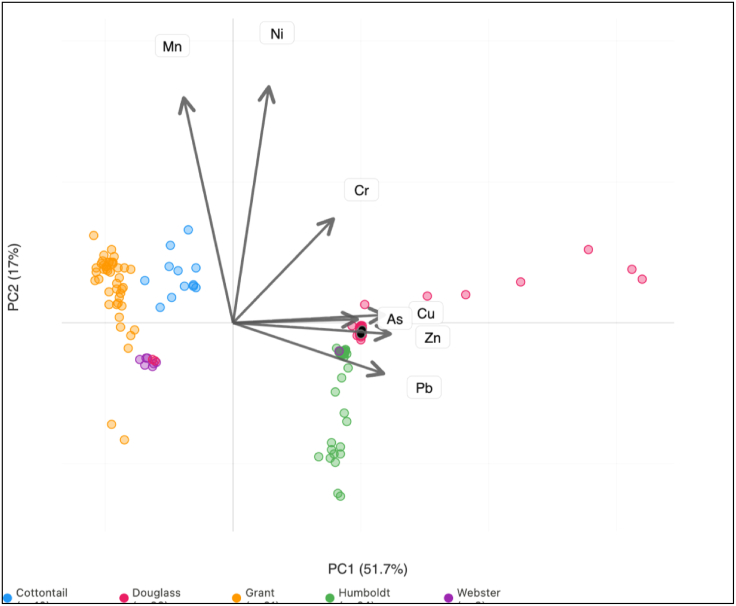
PCA biplot of log-transformed trace element concentrations across five Chicago parks (*n* = 233 samples) Loadings (arrows) represent the direction and strength of each element’s contribution to PC1 (51.7%) and PC2 (17%). Parks are color-coded.

The principal-component analysis (PCA) biplot (Figure 4) illustrates the multivariate structure of trace element concentrations across the five parks. PC1 accounted for 51.7% of the total variance and PC2 for an additional 17%, together explaining 68.7% of the variation in the dataset. Each point represents a soil sample, color-coded by park.

PC1 is driven by strong positive loadings of Pb, Zn, Cu, and As, which collectively define an anthropogenic contamination gradient. Douglass Park samples are displaced farthest along the positive PC1 axis, consistent with its elevated concentrations of these elements. Several Humboldt Park samples also extend into the positive PC1 range, though with greater scatter, reflecting within-park variability. In contrast, Grant, Cottontail, and Webster Park samples cluster tightly at the negative end of PC1, indicating uniformly low concentrations of these metals.

PC2 differentiates elements with a predominantly geogenic signature from the anthropogenic cluster. Mn and Ni load strongly on the positive PC2 axis, nearly orthogonal to the Pb-Zn-Cu group, indicating that these elements vary independently of the main contamination gradient. Cr loads intermediately between PC1 and PC2, suggesting mixed anthropogenic and geogenic contributions. Notably, Douglass Park samples show wide dispersion along both axes, reflecting heterogeneous contamination likely attributable to its construction from stockyard fill materials and surrounding industrial inputs.

### Socio-economic disparities among park communities

The analysis of parks reveals stark socio-economic contrasts across neighborhoods (Table S2). Douglass Park, located in a predominantly minority area (94.7% minority), has the lowest median income ($33,334) and affordable housing prices ($159,000). The community also shows low educational attainment, with 76.1% of residents lacking a degree. Humboldt Park primarily serves a Hispanic-majority population (49.8%) with a moderate median income of $51,105 and slightly higher housing prices ($401,594), though 70.2% of residents lack a degree. Grant Park, characterized by a more diverse population (46.1% minority), has a higher median income of $120,175, significant educational attainment (85.4% with a degree), and median home prices around $403,250. Cottontail Park and Webster Park represent the most affluent neighborhoods, with a similar demographic composition (49.3% minority), high median incomes ($124,558), and expensive housing markets ($392,500 median home price). Both areas also boast the highest levels of educational attainment, with 84% of residents holding a degree. These findings highlight the socio-economic hierarchy impacting communities near urban parks.

### Correlation analysis

**Figure 5:**
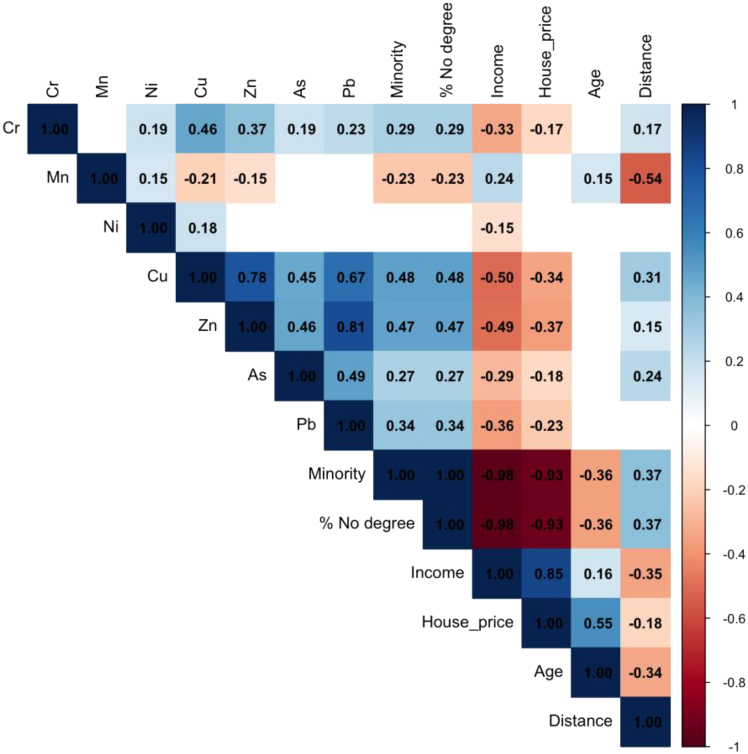
Correlation matrix heatmap of trace elements, socioeconomic variables, park age, and distance from city center across five parks in Chicago (*n* = 233) Colors indicate statistically significant relationships (*p* < 0.05).

The correlation matrix (Figure 5) reveals distinct groupings among trace elements and socioeconomic variables. All correlations reported in the following text are statistically significant (*p* < 0.05). The strongest inter-element correlations occur among Pb, Zn, and Cu: Zn-Pb (r = 0.81), Cu-Zn (r = 0.78), and Cu-Pb (r = 0.67), indicating co-occurrence consistent with shared anthropogenic sources. Arsenic is moderately correlated with Cu (r = 0.45), Zn (r = 0.46), and Pb (r = 0.49), suggesting partial overlap with these contamination sources.

In contrast, Mn is weakly negatively correlated with Cu (r = −0.21) and Zn (r = −0.15), and Ni shows negligible correlations with most other elements, reinforcing the PCA finding that these elements are governed by geogenic rather than anthropogenic processes. Manganese exhibits a strong negative correlation with distance from the city center (r = −0.54), suggesting that Mn concentrations are higher in parks closer to the loop, potentially reflecting differences in parent material or fill composition.

Socioeconomic variables show consistent relationships with the anthropogenic trace elements. Median income is negatively correlated with Cu (r = −0.50), Zn (r = −0.49), Pb (r = −0.36), Cr (r = −0.33), and As (r = −0.29), indicating that lower income neighborhoods tend to have higher contamination levels. Minority population percentage and % without a degree are positively correlated with Cu (r = 0.48), Zn (r = 0.47), Pb (r = 0.34), and Cr (r = 0.29), mirroring the income relationship in the opposite direction. The socioeconomic indicators themselves are highly intercorrelated (Minority-% no degree: r = 1.00; minority-income: r = −0.98; income-house price: r = 0.85), reflecting the entrenched demographic and economic stratification across Chicago neighborhoods. Distance from the city center correlates positively with minority percentage (r = 0.37) and negatively with income (r = −0.35).

## Discussion

### Linking soil properties to socioeconomic factors in Chicago

Contrary to our initial hypothesis, parks located farther from the city center exhibited higher trace element concentrations for most anthropogenic metals. This finding suggests that proximity to the urban core is not the primary driver of contamination in this study and that neighborhood-level factors including industrial history, land use, and socioeconomic characteristics are more explanatory. Urban soils are a source and sink for various environmental contaminants, including Pb, which poses a significant health risk to children. The primary pathway of trace metals in the soils we collected and analyzed in this study is atmospheric deposition. Cations such as Pb and Cd can strongly adsorb to exchange sites on certain clay minerals present in resuspended soil dust. Laidlaw and Filippelli^20^ found that resuspension of Pb-enriched urban soils served as a continued source of bioavailable Pb both outside and inside homes.

Socioeconomic factors show an association with the distribution of trace element contamination across the studied parks. Parks located in higher income areas, such as Grant, Webster, and Cottontail, are characterized by higher median household incomes and property values and generally exhibit lower concentrations of trace elements. In contrast, Douglass and Humboldt Parks, situated in lower income neighborhoods, show consistently higher levels of contamination. These patterns, supported by both PCA and correlation analyses (Figures 4 and 5), indicate that trace element distribution is not random but is structured along socioeconomic gradients. This relationship highlights disparities in environmental quality across urban neighborhoods and suggests that communities with lower socioeconomic status may face a greater burden of soil contamination and health disparities.

Environmental and historical factors, including structural racism,^34^ further explain the observed spatial variability in trace element concentrations across parks. Differences in land-use history, fill materials, and site development likely contribute to the contrasting contamination patterns. For example, Grant Park was constructed primarily from lakefill material, which may have lower initial contamination compared to soils influenced by prolonged urban activity. In contrast, parks such as Douglass and Humboldt may reflect cumulative impacts from historical urban development, including legacy deposition from nearby lead-emitting industries and infrastructure and past land uses. These site-specific factors, combined with heterogeneous soil composition and management practices, likely contribute to the elevated and more variable trace element concentrations observed in these parks.

Soil physicochemical properties across all parks further influence trace element retention and mobility. Soil pH ranged within a near-neutral to slightly alkaline range, with no evidence of extreme acidity or alkalinity, conditions generally favorable for plant growth and reduced metal solubility. EC values indicated non-saline conditions, suggesting limited influence of soluble salts on metal mobility. Organic matter content was relatively high across all parks (6.3%–17.6%), likely reflecting regular organic amendments by park management. Organic matter enhances the formation of stable complexes with metals, reducing their bioavailability, while near-neutral pH further limits metal solubility and mobility.^41^^,^^42^^,^^43^ The combination of near-neutral to alkaline pH and elevated organic matter content suggests a strong capacity for trace element retention in these soils. Under such conditions, metals such as Pb, Cu, and Zn are more likely to adsorb to soil particles and form stable complexes with organic matter, reducing their mobility and immediate bioavailability to plants and humans. However, these same conditions can also promote the long-term accumulation and persistence of trace elements in soil. Reduced mobility limits leaching and transport, leading to prolonged residence times and the preservation of historical contamination signals. As a result, even if bioavailability is lower under current conditions, legacy contaminants may remain in place for extended periods and continue to pose exposure risks through direct soil contact or disturbance.^41^^,^^42^^,^^43^ This creates a dual effect in which soils act both as sinks that stabilize contaminants and as long-term reservoirs that retain legacy pollution.

**Figure 6:**
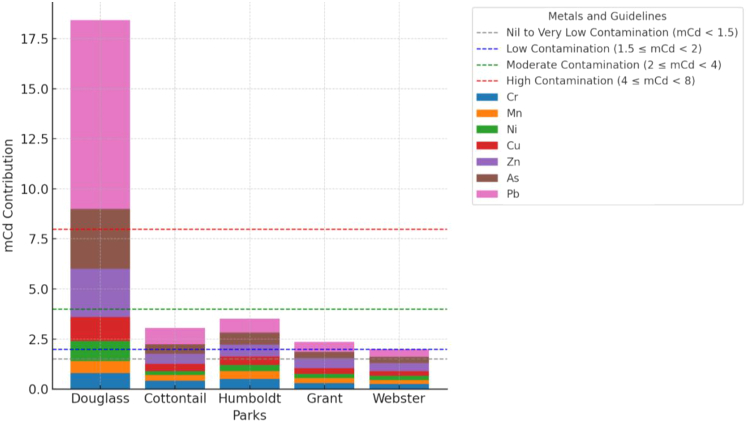
Stacked bar chart showing the contribution of each metal to the overall mCd for the studied park in Chicago based on the mean values

The integrated contamination index (mCd) provides a cumulative perspective on metal contributions across parks (Figure 6). Douglass Park exhibits a markedly higher mCd value, corresponding to a “very high to extremely high” degree of contamination, with Pb, Zn, and Cu contributing most strongly. The elevated contamination at Douglass Park likely reflects its unique construction history; the site was filled with sand and manure from the Chicago Union Stock Yards in the 1870s to transform a marshy wetland into parkland (Chicago Park District, n.d), compounded by its location in North Lawndale, an environmental justice community bordered by active industrial corridors with documented releases of Pb, Cr, and other contaminants (EPA Toxics Release Inventory), as well as legacy deposition from leaded gasoline. In contrast, Grant and Webster Parks show relatively low mCd values, largely within the “low to moderate” contamination range, indicating lower overall contamination. Cottontail and Humboldt Parks fall within the “low to moderate” category but display greater variability. These results underscore substantial spatial differences in contamination across parks and identify Douglass Park as a priority area for intervention due to elevated heavy metal levels, particularly Pb. Within Douglass Park, a localized hotspot was identified in the northwestern sector of the park, where individual samples reached 1,824 mg/kg Pb, 2,259 mg/kg Zn, and 279 mg/kg Cu—concentrations an order of magnitude above the park median. This cluster likely represents a discrete buried contamination source rather than diffuse urban deposition and warrants targeted investigation including depth profiling and historical land use records prior to any soil disturbance or gardening activity in that area.

### Global context and historical legacies of soil contamination in urban parks

Our findings show that trace element concentrations in selected urban park soils vary across locations and align with spatial and socioeconomic gradients, reflecting underlying patterns of urban development. Research across Europe shows variable but generally high levels of Cu, Pb, and Zi in urban parks (Table 3), with especially high values in cities with a legacy of heavy manufacturing like Glasgow and Torino. Cr and Ni exhibited higher concentrations mainly in parks in Italy, pointing to the lasting impacts of industrial legacies.^44^ The significant spatial variability in soil contaminants emphasizes the influence of differing historical land uses. A study in Galway, Ireland, found extremely high Pb concentrations in soil, up to 10,297 mg/kg, at a park previously used as a municipal and industrial waste site.^45^ Similarly, parks in Durham, North Carolina, and around a nature preserve in Chicago showed significant Pb contamination, attributed to historical industrial activities and inadequate waste management practices (Bihari et al.^46^). This pattern is consistent across various studies, including those in Beijing, where soil contamination with Cu and Pb was significant, particularly in densely populated districts.^47^ Collectively, these findings underscore that urban park soils are not inherently clean refuges but rather archives of past land use, and their trace element profiles must be evaluated in the context of site-specific industrial, municipal, and developmental histories.

**Table 3:** Urban soil trace elements (in mg/kg) in parks worldwide

City	N	Pb	As	Cu	Mn	Ni	Zn	Cr	Source
Chicago, USA	233	146	14	59	405	36	208	79	present study
Grand Forks, ND, USA	30	17	9	17	818	24	80	24	Saleem et al.^48^
Brno, Czech Republic	61	27	–	17	–	–	59	–	Brtnický et al.^49^
Galway, Ireland∗	200	328	18	88	–	–	302	–	Carr et al.^45^
Palermo, Italy	70	202	–	63	519	–	138	34	Manta et al.^23^
Madrid, Spain	40	22	7	14	249	–	50	17	De Miguel et al.^50^
Seville, Spain	31	137	–	68	471	22	145	39	Madrid et al.^44^
Çanakkale, Turkey	42	18	–	28	475	21	58	21	Parlak et al.^51^
Peshawar, Pakistan	85	22	–	20	–	64	78	35	Khan et al.^52^
Beijing, China	–	36	12	35	–	27	146	64	Liu et al.^53^
Moscow, Russia∗	19	15	5.4	17	493	19	70	52	Shvetsova et al.^54^

Note: ∗median.

### Recommendations for best management practices

The guideline for Pb in residential soils is 200 mg/kg,^55^ while there is no safe level for children’s play areas due to the serious risks associated with even minimal exposure, which can lead to cognitive decline, developmental delays, and behavioral changes in children.^56^ These findings are supported by accumulating evidence showing that children are particularly vulnerable to soil contaminants, as they are more likely to ingest soil particles during play, and their developing bodies are more susceptible to the toxic effects of heavy metals.^57^

Given these variations and the potential health risks, it is crucial to conduct soil testing before planting edible crops in urban or peri-urban gardens. Appropriate remediation strategies such as soil amendments (e.g., composts, biochar, and phosphates) or raised beds with clean imported soil should be implemented if contaminant levels exceed these thresholds.^58^ Soil amendments can reduce bioavailability of metals, while raised beds can effectively prevent direct contact with contaminated soils, reducing the risk of human exposure.^58^ These proactive measures not only mitigate health risks but also foster safer urban gardening practices, supporting healthier and more productive green spaces.

According to the Groundwork Atlanta suitability guidelines (Table 4), Webster Park levels were found to fall under Action Level 1, which suggests that it is safe to garden in the native soil here, except for vegetables with deep roots (such as beets, carrots, radishes, turnips, tomatoes, and sweet potatoes) unless in a raised bed. Alternatively, Cottontail and Grant Parks fall under Action Level 2, which means they are safe for urban gardening with contact to native soil, with restrictions, such as an application of compost and growing nut and fruit trees. Otherwise, raised beds are necessary, and they do not recommend planting anything with deep roots—even in raised beds. Humboldt and Douglass Parks fall under Action Level 3, which means they are not safe for urban gardening with contact to native soil. Groundwork Atlanta suggests the necessity for extensive soil amendments or the establishment of raised beds to prevent direct contact with contaminated soil. It should be noted that these classifications represent park-wide assessments based on overall concentration ranges. Given the substantial within-park variability documented in this study, particularly at Douglass Park, where Pb ranged from 19 to 1,824 mg/kg, site-specific soil testing at the plot level is strongly recommended before any gardening activity is initiated, regardless of park-wide classification. Such interventions are essential to ensure that urban agriculture initiatives can safely contribute to food security and community resilience in urban environments. Clean organic matter, such as compost and manure, can aid soil rehabilitation. It is recommended to reduce dust by covering bare soil with groundcover or mulch, peel root vegetables before consumption, and focus on crops with shallow roots for safety.

**Table 4:** Groundwork Atlanta’s urban gardening action levels are based on trace element concentration

Metal	Action Level 1^a^ (caution) (mg/kg)	Action Level 2^b^ (Restrictions) (mg/kg)	Action Level 3^c^ (eliminate) (mg/kg)
Arsenic	2.7	11	110
Chromium	5	390	630
Lead	34	100	340

Gardening recommendations.

a Level 1: root veg: OK; shallow roots: OK; deep roots: raised bed; composting: OK; mushrooms: OK; grains: OK; berries: OK; fruiting vines: OK; nut/fruit trees: OK.

b Level 2: root veg: raised bed; shallow roots: raised bed; deep roots: no; composting: OK; mushrooms: raised bed; grains: raised bed; berries: raised bed; fruiting vines: raised bed; nut/fruit trees: OK.

c Level 3: eliminate use for food production.

To effectively monitor urban parks, prevent human exposure to contaminants, and enhance urban agriculture in Chicago, a multifaceted approach is essential. Research recommendations include comprehensive soil mapping of trace element contamination,^59^ studies on bioavailability of trace elements in urban soils,^60^ investigation of cost-effective remediation techniques,^61^ establishment of long-term monitoring programs,^62^^,^^63^ and health impact assessments correlating soil contamination with health outcomes.^64^

### Socioeconomic and environmental justice considerations

The analysis of Chicago’s parks and neighborhoods reveals a pattern of environmental injustice, with lower income and predominantly minority areas facing disproportionate environmental burdens. Parks in these communities, such as Douglass Park in an African American neighborhood and Humboldt Park in a diverse area with a large Hispanic population, show significantly higher contamination levels, particularly for trace elements like Pb and Cu, compared to parks in wealthier areas.

The Chicago Department of Health (2023) has quantified this inequality in the Chicago Cumulative Impact Assessment, identifying nearly 30% of the city’s census tracts as environmental justice neighborhoods, with South and West Side areas being disproportionately impacted by higher heat-related illnesses, industry pollution, increased amounts of ground-level ozone and particulate matter, flooding, lack of green space, higher density of buildings and pavement, as well as historic disinvestment.^65^ Douglass Park is situated in the North Lawndale Community Area, which was classified as an environmental justice neighborhood, along with the Humboldt Park neighborhood. This pattern suggests that Chicago’s industrial legacy and socioeconomic factors have contributed to these unevenly distributed environmental burdens, raising concerns about equitable access to safe, healthy green spaces.

In response to these challenges, Chicago has launched several initiatives to address environmental inequities. The city planted over 11,000 trees in 2023, focusing on high-need areas to improve air quality and repair tree canopy.^66^ There is a goal to plant an additional 15,000 trees in 22 community areas with the lowest tree canopy. Additionally, the city is developing green alley projects and a sustainable community air monitoring strategy in partnership with environmental justice organizations.^67^

Despite these efforts, significant challenges remain. Historical zoning practices and development patterns have concentrated environmental burdens in certain communities, particularly on the South and West sides.^67^ To address these deep-rooted issues, the city has developed an Environmental Justice Action Plan and is working on a cumulative impact ordinance. These policy measures aim to reform land use, zoning, and environmental enforcement practices to better protect vulnerable communities.^65^ Implementing comprehensive soil testing before any urban agriculture is initiated, particularly in parks serving lower income communities, is essential. Where contamination exceeds safe thresholds, remediation strategies including the addition of clean soil, organic amendments, and raised beds should be adopted. Urban planning and policy must prioritize environmental justice by integrating soil health into green infrastructure decisions and increasing investment in park maintenance in underserved areas.

### Limitations of the study

This study is subject to several limitations. First, trace element concentrations were measured using pXRF, which quantifies total concentrations but does not distinguish between chemical species or bioavailable fractions, potentially leading to overestimation of risk for elements such as chromium. Second, soil sampling was restricted to the 0–15 cm depth and targeted areas suitable for gardening, which may not fully represent spatial heterogeneity across entire parks or deeper soil profiles. Given Chicago’s extensive pre-1978 housing stock and history of heavy industry, lead concentrations and, by extension, other trace metal concentrations vary substantially across the city.^67^ Third, while correlations between contamination and socioeconomic factors were observed, causal relationships cannot be established due to potential confounding factors such as land-use history and management practices. Finally, risk assessments were based on screening-level comparisons rather than site-specific exposure or bioavailability data and thus should be interpreted as conservative estimates.

Future research should expand the spatial scope of sampling, investigate bioavailability and speciation of key contaminants, particularly Cr and Pb, and extend monitoring to emerging contaminants including microplastics and per- and polyfluoroalkyl substances (PFAS or “forever chemicals”). Long-term monitoring programs and health impact assessments linking soil contamination to measured exposure outcomes in park users would significantly strengthen the evidence base for targeted remediation in Chicago and other legacy industrial cities.

## Resource availability

### Lead contact

Further information and requests for resources should be directed to and will be fulfilled by the lead contact, Anna A. Paltseva (apaltsev@purdue.edu).

### Materials availability

This study did not generate new unique reagents. Soil samples were collected from public urban parks and were consumed during analysis; no physical samples are available for distribution.

### Data and code availability

• The dataset generated in this study, including trace element concentrations and soil physicochemical properties, is available at https://data.mendeley.com/datasets/n97b86zkzf/1. Statistical analyses were conducted in R (v.4.2.3) using standard, widely established packages and functions.
• No custom code or novel algorithms were developed.
• Any additional information required to reanalyze the data reported in this paper is available from the lead contact upon request.

## Acknowledgments

We extend our sincere gratitude to the 10.13039/100009171USDA NRCS for their assistance, training, guidance, and funding via 10.13039/100000199USDA Cooperative Agreement award no. NR223A750025C008. We also wish to graciously thank Nicholas Miller from the University of Louisiana at Lafayette for his meticulous preparation and testing of soil samples.

## Author contributions

E.M.J.: data curation, formal analysis, investigation, visualization, and writing – original draft; H.H.: data curation, investigation, and visualization; J.M.: conceptualization, validation, methodology, and writing – review and editing; V.S.: visualization and formal analysis; A.A.P.: conceptualization, funding acquisition, methodology, project administration, resources, supervision, and writing – review and editing.

## Declaration of interests

The authors declare no competing interests.

## Declaration of generative AI and AI-assisted technologies in the writing process

During the preparation of this work, the authors used ChatGPT to improve readability and language of the manuscript. After using this service, the authors reviewed and edited the content as needed and take full responsibility for the content of the published article.

## STAR★Methods

### Key resources table

**Table:** 

REAGENT or RESOURCE	SOURCE	IDENTIFIER
**Software and algorithms**		

RRID:SCR_000432 (RStudio) and RRID:SCR_001905 (R Project). RStudio 2024.09.0+375 (RStudio Team 2016) and R 4.2.3 (R Core Team 2018)	R Core Team	https://cran.r-project.org/

**Other**		

Thermo Scientific Niton XL3t 955 Ultra pXRF analyzer	Thermo Fisher Scientific	https://www.thermofisher.com/
Certified reference materials (SRM 2710a, 2711a, 2704, 2709a)	NIST SRM	https://www.nist.gov/srm
Hanna HI5521-01 pH meter	Nannah Instruments	https://hannainst.com/
YSI 3100 conductivity meter	YSI Xylem	https://www.ysi.com/

### Experimental model and study participant details

Omitted as our study does not involve biological models.

### Method details

#### Environmental setting and study sites: Physiography, geology, and soils

Chicago, Illinois is the third largest city in the United States, and the largest city in the state of Illinois. Located in Cook County, Chicago is home to 2.7 million residents.^68^ Chicago is part of the Wheaton Morainal Country and the Chicago Lake Plain (CLP) subsections of the Great Lakes Section, which is a subdivision of the Central Lowland Province.^69^^,^^70^ The CLP is a broad, low-slope area that formed the floor of Glacial Lake Chicago, a prehistoric proglacial lake that represented an early ancestral stage of modern Lake Michigan. Glacial Lake Chicago occupied the southern Lake Michigan basin where meltwater was impounded between the retreating ice front and arcuate moraines that ring the basin’s southern margin. During late Wisconsinan deglaciation, roughly 14,000 to 11,000 years BP, the areal extent and depth of this ancestral lake fluctuated as the glacier retreated and outlets evolved. Subsequent Holocene lake stages, including the Nipissing and Algoma high stands, brought Lake Michigan toward its present configuration, with near-modern water levels established by around 2,500 years BP.^71^^,^^72^

Surficial geomorphic features in the county include depositional moraines, outwash plains, valley trains, filled lake basins, river flood plains, and sand dunes.^70^^,^^72^ Soils formed in parent materials deposited during Wisconsinan glaciation. Parent materials included surficial deposits of till, lacustrine sediment, outwash, beach deposits, and alluvium. Parent materials were distributed by ice, water, and wind. Till was deposited directly by glacial ice, while meltwater streams deposited outwash sediment. Shallow lakes formed when drainage from ice fronts was blocked, resulting in the deposition of fine-grained sediments. Larger lakes, such as Lake Chicago, were characterized by sandy beach ridges along their perimeter.

Soils in the CLP supported pre-settlement vegetation communities including dry and mesic prairie, forest and savanna, wet prairie, and marsh.^73^ Native glacial lakebed soils are characterized by thick, dark topsoils, with underlying dense clay horizons, formed in wet prairies. Along ancient shorelines in the CLP, drier sand beaches have thin and moderately dark topsoils formed under prairie and forest vegetation. Urbanization has heavily altered the landscape, raising the land surface through filling and creating soils more suited for construction than plant growth due to disrupted water cycles.^74^

The city of Chicago has undergone extensive anthropogenic modification since the 1830s resulting in a highly altered urban landscape, or anthroscape.^75^ Large-scale engineering activities reshaped the natural environment through the filling of shallow sloughs adjacent to the Chicago River, straightening of river channels, excavation of canals, and extensive infilling along the Lake Michigan shoreline to create new land for beaches and parks. In the central business district, known locally as the “Loop”, land elevation was raised by up to three meters to promote drainage and improved sewage management.^75^ Much of the lakefront parkland was constructed using dredged sediments from Lake Michigan, construction debris, and materials generated during major urban events, including the Chicago Fire of 1871. As the result, the soils within these parks have been developed in human-altered and human-transported materials, consistent with the soil series described in Table 1. Consequently, undisturbed native soils are largely absent, and measured trace element concentrations reflect both historical input and ongoing anthropogenic influences rather than baselines geogenic conditions.

This study focused on five Chicago parks, each chosen for their distinctive characteristics and locales as depicted in Figure 1 and Table 1. The parks include Douglass Park, Humboldt Park, Grant Park, Cottontail Park, and Webster Park. Grant Park, located in Chicago’s central business district in the east and spanning 312.98 acres, is particularly of interest due to its high traffic, dense urban setting, and frequent construction, potentially influencing contamination patterns. Douglass Park, occupying 162 acres on the West Side, has transformed from a marshy site to a park equipped with modern facilities like mini golf courses, soccer fields, and swimming pools, alongside its demographic setting in a majority African American community, makes it a focal point for studying environmental justice and potential legacy contamination.^76^^,^^77^ Similarly, Humboldt Park, also on the West Side, covers 197.26 acres with its historical environmental burdens and the park’s role in hosting high-traffic events were central to its selection for the study. Cottontail Park and Webster Park, both located in the Near South Side community and measuring 2.35 acres and 1.20 acres respectively, are surrounded by fewer large-scale events and constructions compared to the other parks. Their sizes, lack of construction, and low-traffic providing a contrast to the more developed and event-heavy parks like Grant Park (Chicago Park District, n.d.).

#### Soil sampling

Sampling plots were selected based on criteria that included open spaces free from construction, located away from roads, and not heavily shaded by trees. Soil samples were collected at a depth of 0–15 cm (standard practice because it represents the primary rooting zone for most vegetables and shallow-rooted crops) using a garden shovel. Each soil sample consisted of a composite of three subsamples collected within approximately 1 m of a single point, arranged in a grid-like pattern within each designated plot. Sampling followed an approximate 10 m grid spacing.

The coordinates of each sampling point were recorded for subsequent spatial analysis. Depending on plot size, between 6 and 18 composite samples were collected (smaller plots required fewer samples, while larger plots required more to capture spatial variability). All samples were placed in labeled Ziploc bags for transport and storage. In total, 233 soil samples were collected across all study parks.

#### Sample preparation

Soil samples were oven-dried at 105°C for 24 h to achieve constant weight. Dried samples were ground using a mortar and pestle and sieved to <2 mm particle size. Prepared samples were stored in low-density polyethylene bags prior to analysis.

#### Trace element analysis (pXRF)

Laboratory analyses were performed in the Delta Urban Soil Laboratory at the University of Louisiana at Lafayette. Soil samples were oven-dried at 105°C for 24 h, ground with a mortar and pestle, and sieved to a particle size of <2 mm with a sieve #10. The prepared samples were placed in low-density polyethylene bags and analyzed using a handheld portable X-ray fluorescence (pXRF) analyzer (Thermo Niton XL3t 955 Ultra). Prior to sample analysis, polyethylene bags were scanned to confirm that the concentrations of the target elements (Pb, As, Cr, Cu, Mn, Ni, and Zn) were below the detection limit.

Each sample was analyzed for 90 s using the pXRF analyzer’s “Soil Mode.” Standard reference materials (SRM 2710a, SRM 2711a, SRM 2704, and SRM 2709a) were used with each batch of samples, and the experimental values were within 100 ± 10% of the certified values. The XRF’s detection limits for the trace elements analyzed were as follows: 8 mg/kg for Pb, 7 mg/kg for As, 30 mg/kg for Cr, 15 mg/kg for Cu, 65 mg/kg for Mn, 30 mg/kg for Ni, and 12 mg/kg for Zn.^78^

#### Soil property measurements

Soil organic matter (SOM) content was quantified as a percentage of the sample’s dry weight using the loss on ignition (LOI) method. After oven-drying the samples at 105°C to achieve constant weight, they were combusted at 550°C for 20 min to eliminate organic material, following grinding and sieving to a particle size of <2 mm (Paltseva et al., 2018). The soil pH was measured in a 1:1 soil-to-water mixture with Hannah HI5521-01Meter, while electrical conductivity (EC) was determined using a 1:2 soil-to-water mixture with YSI 3100 Conductivity Instrument, with values reported in microSiemens per centimeter (μS/cm). Quality control was ensured using SRMs (buffer solutions with pH of 4.01, 7.01, and 10.01; conductivity standard solution of 1413 μS/cm) and probe calibration with deionized water prior to each analytical session.

#### Socioeconomic data

To assess the relationship between neighborhood socioeconomic characteristics and trace element contamination across the five study parks, data on median income, racial demographics, and educational attainment were obtained from the Chicago Metropolitan Agency for Planning Community Data Snapshots, based on U.S. Census Bureau American Community Survey 2018–2022 five-year estimates.^76^ Data was reported at the Chicago Community Area level: Douglass Park falls within North Lawndale (CCA 29), Humboldt Park within the Humboldt Park community area (CCA 23), Grant Park within The Loop (CCA 32), and Cottontail and Webster Parks within Near South Side (CCA 33). Median home prices were obtained from Zillow (accessed 2024) based on Zillow Home Value Index to supplement housing market context for each community area.

#### Statistical and geospatial analyses

A principal component analysis (PCA), correlation matrix, boxplots, and scatterplots were generated using RStudio 2024.09.0+375 (RStudio Team 2016) and R 4.2.3 (R Core Team 2018) to evaluate relationships between trace elements (log-transformed Cr, Mn, Ni, Cu, Zn, As, Pb) and socioeconomic factors (race, income, housing prices, and education) in the parks’ neighborhoods. Dunn’s test non-parametric post-hoc test following a Kruskal-Wallis test, was used with Bonferroni correction to compare metal concentrations between parks, ensuring robust pairwise comparisons for non-normally distributed data with unequal sample sizes.

#### Modified contamination degree (mCd)

The Modified Contamination Degree (mCd) is an index used to assess the overall contamination of a site by multiple trace elements.^79^ It provides insight into the cumulative pollution load by evaluating the ratio of observed concentrations to regional background values. The mCd index is calculated using the formula.

$$mC_{d}=\frac{\sum_{i=1}^{i=n}C_{f}^{i}}{n}$$

where n = number of analyzed elements; i = ith element (or pollutant); cf. = Contamination factor.

#### The Contamination Factor (CF) for each metal is given by

$${CF}_{i}=\frac{C_{i}}{C_{background}}$$

where C_*i*_ = Concentration of metal *i* in the soil sample; *C*_*background*_ = Geochemical background concentration of metal *i*. We used the mean concentration of each trace metal at each park as C_*i*_, consistent with the index formulation of^79^ We note that mean values at Douglass Park are influenced by a localized hotspot and may overestimate park-wide contamination relative to median values reported elsewhere in this study. The *C*_*background*_ values are reported in Table 2, according to Zhang and Frost (2002).

The mCd index gives a value that is the average of the contamination factors across all assessed metals, providing an overall contamination assessment for the site. The interpretation of mCd values is based on categorizing the degree of contamination.^79^

• mCd <1.5 Nil to very low degree of contamination
• 1.5 ≤ mCd <2 Low degree of contamination
• 2 ≤ mCd <4 Moderate degree of contamination
• 4 ≤ mCd <8 High degree of contamination
• 8 ≤ mCd <16 Very high degree of contamination
• 16 ≤ mCd <32 Extremely high degree of contamination
• mCd ≥32 Ultra high degree of contamination

#### Groundwork Atlanta urban soil suitability guidelines

To understand suitability of park area to establish gardening practices, Groundwork Atlanta’s suitability table^80^ was used as a guideline (see Table 4). Although developed for the City of Aglanta’s Grows-A-Lot program, the action level thresholds are not calibrated to local soil properties. They are derived from the Toronto Public Health Urban Gardening Soil Screening Values and U.S. EPA guidelines for reducing exposure to contaminated soils in urban agriculture settings^81^^,^^82^^,^^83^ As such, these thresholds represent health-based screening values applicable across soil types. We adopted this framework for its practical, tiered structure linking soil test results directly to gardening recommendations, rather than for any soil-specific calibration. The Aglanta Grows-A-Lot recommendations emphasize the importance of considering historical land use and soil testing for potential contamination when establishing community gardens or farms.

Based on soil testing results, recommendations are provided for the growth of various crops, categorized into Action Levels 1, 2, and 3. These levels, in ascending order, reflect different levels of caution. Action Level 1 suggests caution in contact with native soil, Action Level 2 imposes restrictions, and Action Level 3 advises eliminating contact with soil through use of raised beds or containers. Recommendations also include amending soil with clean soil and organic matter, as well as practicing good gardening hygiene. Additionally, information is provided on what types of crops are suitable to grow at each Action Level.

### Quantification and statistical analysis

Statistical analyses were performed using R (v4.2.(3) and RStudio (v2024.09.0+375). Trace element data were log-transformed to improve normality and reduce skewness prior to multivariate analysis. Differences in trace element concentrations among parks were evaluated using the Kruskal–Wallis non-parametric test. Pairwise comparisons were conducted using Dunn’s post hoc test with Bonferroni correction. Statistical significance was defined as *p* < 0.05.

Principal component analysis (PCA) was performed on log-transformed, *Z* score standardized trace element concentrations (Pb, Zn, Cu, As, Cr, Mn, Ni) to evaluate multivariate contamination structure across parks. Correlation analyses were used to assess relationships between trace elements, soil properties, and socioeconomic variables; only statistically significant correlations (*p* < 0.05) are reported. All 233 samples were included in statistical analyses. Values below the instrument detection limit (<DL) were retained in the dataset and reported as such; these values were excluded from log-transformation procedures and PCA to avoid undefined values.
