# Supplementary material for: Legacy contamination in Chicago urban park soils: Spatial patterns, socioeconomic disparities, and implications for urban agriculture
Source: iScience. 2026 Jun 11;29(6):116298. doi: 10.1016/j.isci.2026.116298 (PMC13267565; doi:10.1016/j.isci.2026.116298)
Supplement: Document S1. Figures S1–S6 and Table S1 [file mmc1.pdf]

iScience, Volume *29*

Supplemental information

Legacy contamination in Chicago urban park soils: Spatial patterns, socioeconomic disparities, and implications for urban agriculture

Eriell M. Jenkins, Holly Heafner, James Montgomery, Victoire Soumano, and Anna A. Paltseva

Supporting Information

Table S1. Summary statistics for trace element concentrations and soil properties measured in each park in Chicago. <DL means below the detection limit of pXRF.

Cottontail Park (n=12)

	Cr	Mn	Ni	Cu	Zn	As	Pb	pH	EC,	OM,
				mg/kg					µS/cm	%
**median**	67	485	37	40	134	12	95	7.47	341	11
**mean**	68	474	37	41	146	10	98	7.51	329	10.5
**st. dev.**	6	89	9	6	42	5	35	0.15	104	2.8
**min**	61	362	23	33	103	<DL	52	7.29	156	6.3
**max**	83	677	49	50	268	18	174	7.8	480	14.4

Grant Park (n=43)

	Cr	Mn	Ni	Cu	Zn	As	Pb	pH	Salts,	OM,
				mg/kg					µS/cm	%
**median**	74	458	34	41	145	11	88	7.68	164	8.8
**mean**	72	461	31	43	153	10	120	7.64	175	9.2
**st. dev.**	16	141	18	17	48	5	193	0.29	58	1.9
**min**	<DL	81	<DL	<DL	76	<DL	4	7.17	72	6.5
**max**	101	823	58	120	259	28	1311	8.26	390	13.8

Humboldt Park (n=78)

	Cr	Mn	Ni	Cu	Zn	As	Pb	pH	Salts,	OM,
				mg/kg					µS/cm	%
**median**	79	364	41	59	181	13	121	7.34	208	9.0
**mean**	80	356	37	62	198	14	137	7.38	223	9.2
**st. dev.**	9	50	15	14	52	6	55	0.18	86	1.7
**min**	61	214	<DL	38	87	<DL	39	7.04	78	6.5
**max**	101	492	65	111	396	28	417	8.01	443	12.7

Webster Park (n=9)

	Cr	Mn	Ni	Cu	Zn	As	Pb	pH	Salts,	OM,
				mg/kg					µS/cm	%
**median**	70	468	32	36	111	9	62	7.52	363	8.8
**mean**	70	512	28	36	112	9	61	7.51	359	9.0
**st. dev.**	7	77	13	9	9	2	13	0.06	78	2.3
**min**	59	457	<DL	22	98	7	39	7.40	265	6.6
**max**	82	696	44	51	125	12	81	7.59	482	12.8

Douglass Park (n=91)

	Cr	Mn	Ni	Cu	Zn	As	Pb	pH	Salts,	OM,
				mg/kg					µS/cm	%

**median**	81	400	40	63	207	14	133	7.56	274	10.0
**mean**	84	402	39	69	260	16	181	7.53	283	10.6
**st. dev.**	15	47	14	37	300	7	264	0.28	119	2.7
**min**	67	293	<DL	22	79	9	19	6.86	95	6.5
**max**	149	526	65	279	2259	53	1824	8.22	702	17.6

Table S2. Demographic and economic overview of park communities in Chicago. Data on median income, racial and ethnic composition, and educational attainment were obtained from the Chicago Metropolitan Agency for Planning (2018-2022), while median home prices were sourced from Zillow (2024).

Park	Black (%)	Asian (%)	Hispanic (%)	White (%)	Mixed/ Other (%)	Minority (%)	No Degree (%)	With Degree (%)	Median Income ($)	Median House Price ($)
Douglass	77.7	0.2	14.6	5.3	2.2	94.7	76.1	23.9	33334	159000
Cottontail	23.8	15.2	5.3	50.7	5	49.3	16	84	124558	392500
Grant	8.1	22.2	11.5	53.9	4.3	46.1	14.6	85.4	120175	403250
Humboldt	34.6	2.2	49.8	11.8	1.6	88.2	70.2	29.8	51105	401594
Webster	23.8	15.2	5.3	50.7	5	49.3	16	84	124558	392500

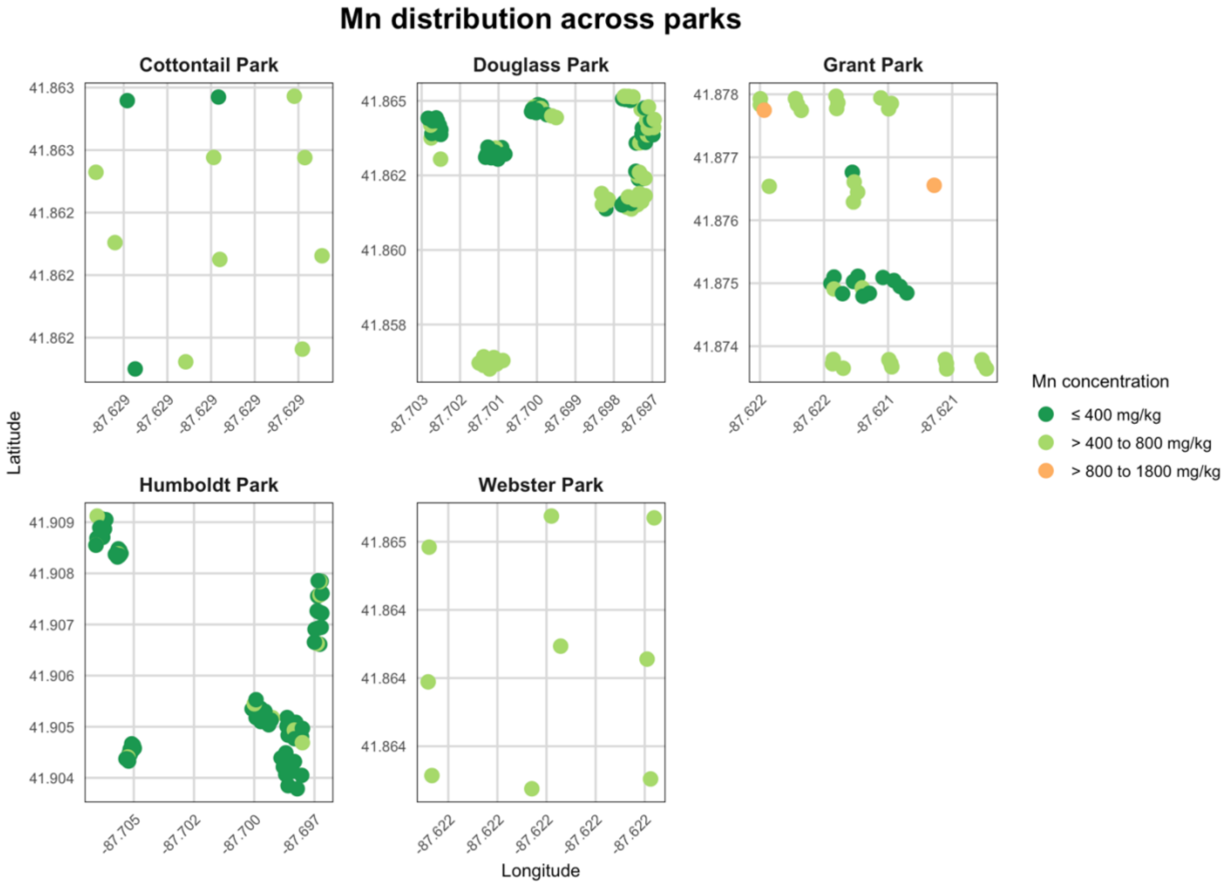

Figure S1. Spatial distribution of Manganese across five urban parks in Chicago. Points represent individual soil sampling locations.

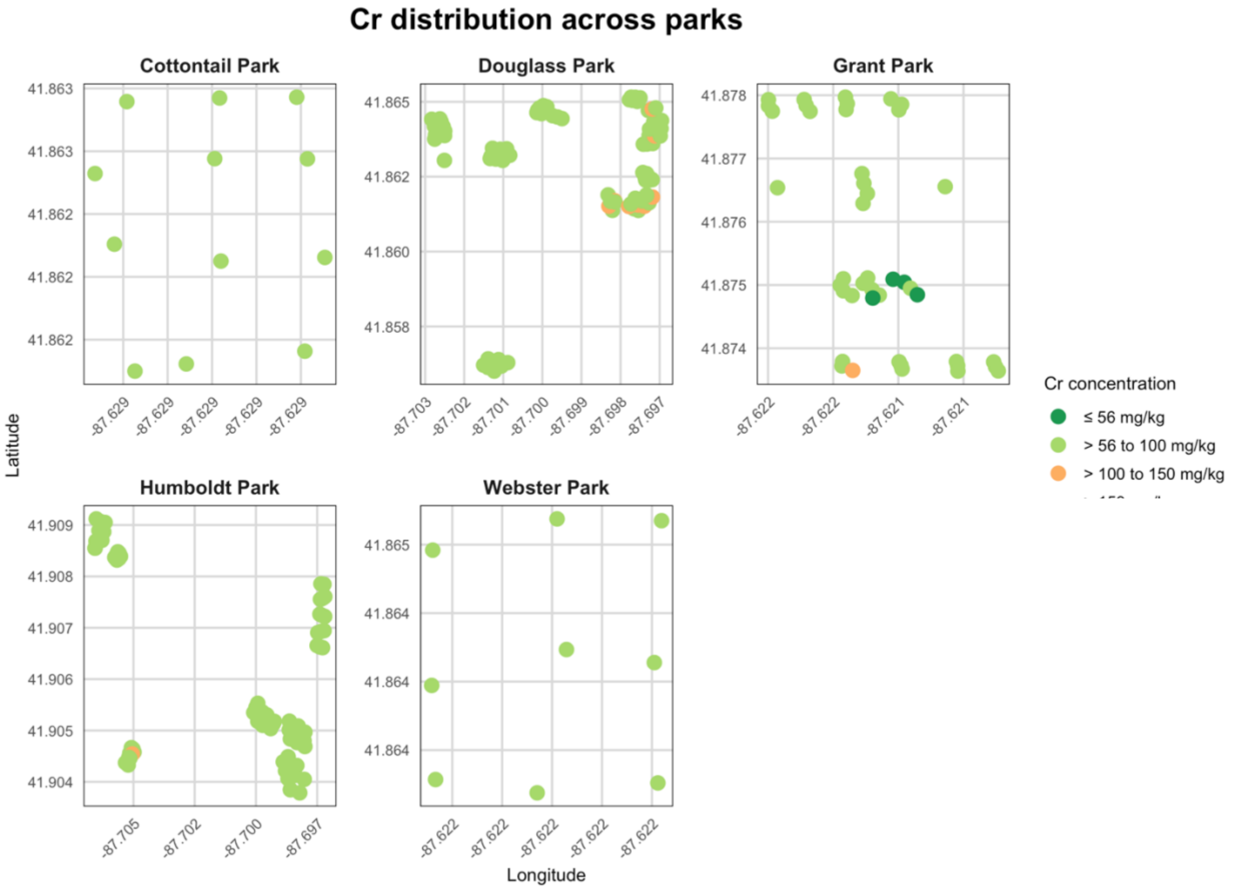

Figure S2. Spatial distribution of Chromium across five urban parks in Chicago. Points represent individual soil sampling locations

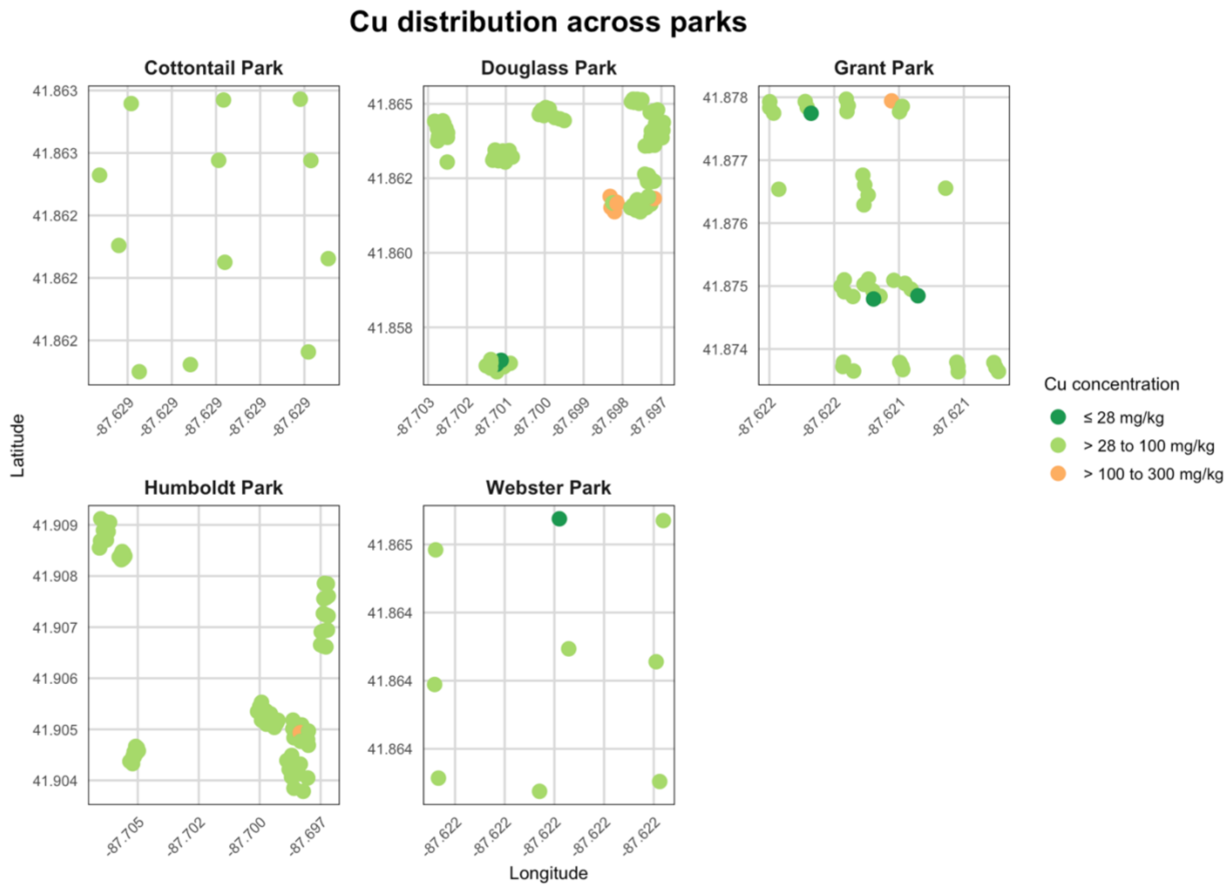

Figure S3. Spatial distribution of Copper across five urban parks in Chicago. Points represent individual soil sampling locations

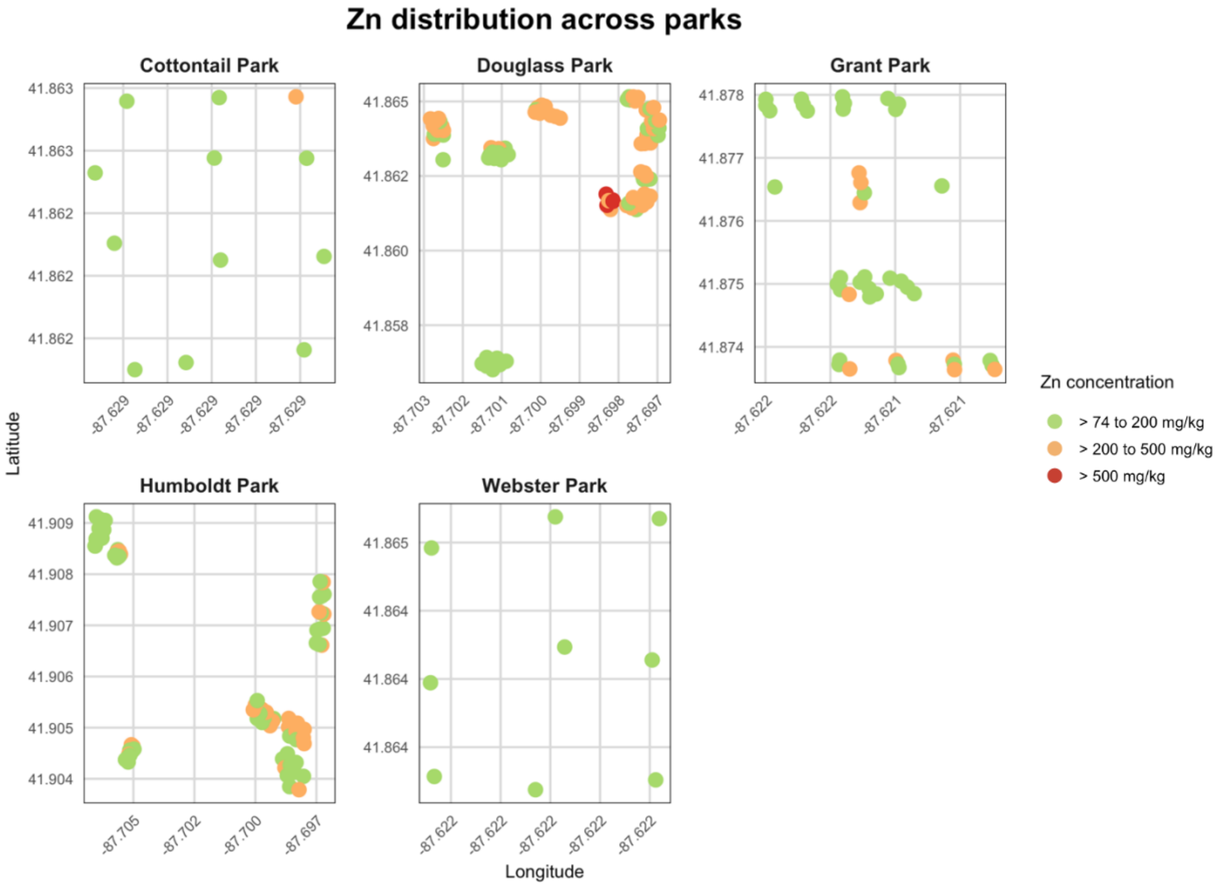

Figure S4. Spatial distribution of Zinc across five urban parks in Chicago. Points represent individual soil sampling locations

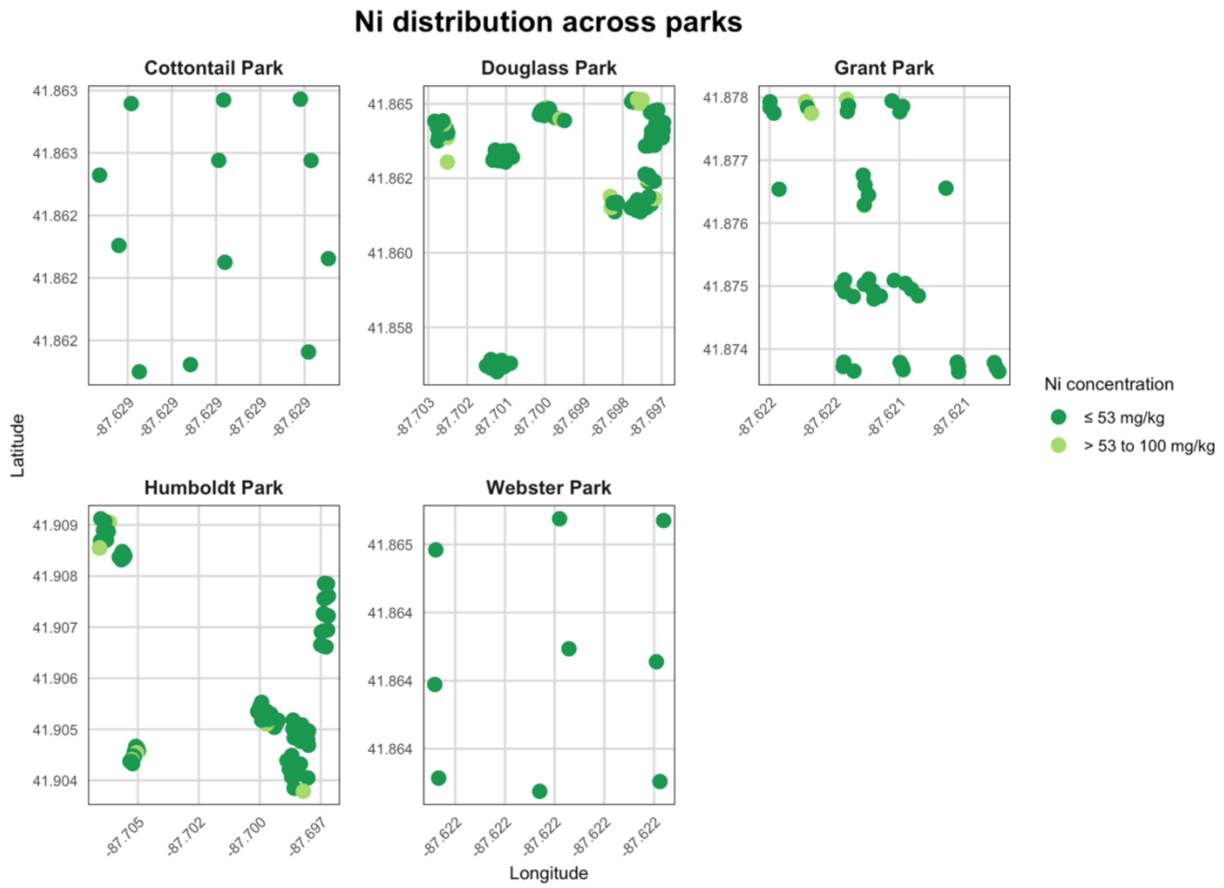

Figure S5. Spatial distribution of Nickel across five urban parks in Chicago. Points represent individual soil sampling locations

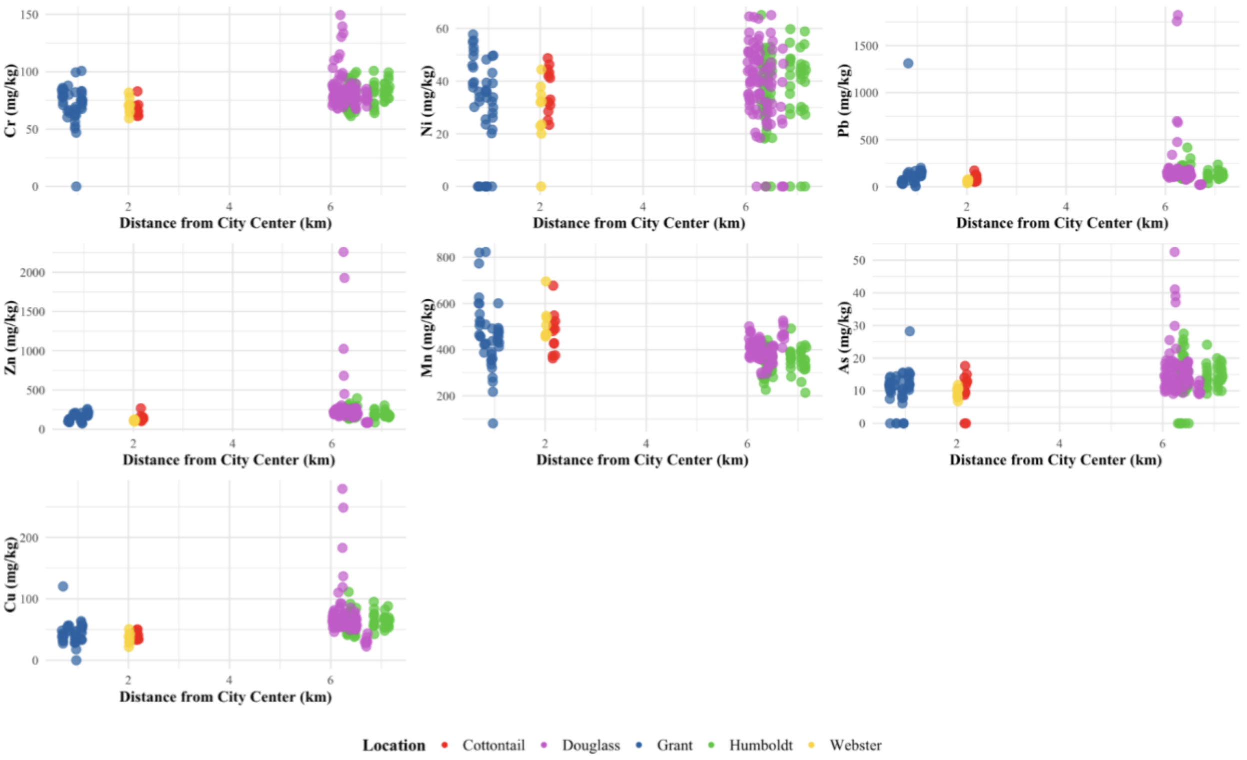

Figure S6. An overview of the trace element variation between parks as a function of the samples' distance from the city center.
